# Home Food Environment Changes and Dietary Intake during an Adolescent Behavioral Weight Loss Intervention Differ by Food Security Status

**DOI:** 10.3390/nu14050976

**Published:** 2022-02-25

**Authors:** Elizabeth L. Adams, Laura J. Caccavale, Jessica Gokee LaRose, Hollie A. Raynor, Melanie K. Bean

**Affiliations:** 1 Department of Exercise Science, University of South Carolina, 921 Assembly St., Columbia, SC 29208, USA; elizabeth-adams@sc.edu; 2 Department of Pediatrics, Children’s Hospital of Richmond at Virginia Commonwealth University, Box 980140, Richmond, VA 23298, USA; laura.caccavale@vcuhealth.org; 3 Department of Health Behavior and Policy, School of Medicine, Virginia Commonwealth University, Box 980430, Richmond, VA 23298, USA; jessica.larose@vcuhealth.org; 4 Department of Nutrition, University of Tennessee, 1215 W. Cumberland Ave., Knoxville, TN 37996, USA; hraynor@utk.edu

**Keywords:** food security, diet, adolescent, obesity, randomized clinical trial

## Abstract

Behavioral weight loss (BWL) for pediatric obesity includes guidance on improving the home food environment and dietary quality; yet food insecurity presents barriers to making these changes. This study examined if home food environment, dietary quality, energy intake, and body weight changes during adolescent obesity treatment differed by food security status, and if changes in the home food environment were associated with changes in dietary quality and energy intake by food security status. Adolescents (*n* = 82; 13.7 ± 1.2 years) with obesity participated in a 4-month BWL treatment. Food insecurity, home food environment (Home Food Inventory [HFI]), dietary quality (Healthy Eating Index [HEI]), energy intake, and body mass index (BMI) were assessed at baseline and post-treatment. A reduced obesogenic home food environment and improved dietary quality were observed for food secure (ps < 0.01), but not insecure households (ps > 0.05) (mean difference, HFI: −6.6 ± 6.4 vs. −2.4 ± 7.4; HEI: 5.1 ± 14.4 vs. 2.7 ± 17.7). Energy intake and BMI decreased for adolescents in food secure and insecure households (ps < 0.03) (mean difference; energy intake: −287 ± 417 vs. −309 ± 434 kcal/day; BMI: −1.0 ± 1.4 vs. −0.7 ± 1.4). BWL yielded similar reductions in energy intake and body weight yet did not offer the same benefits for improved dietary quality and the home food environment for adolescents with food insecurity.

## 1. Introduction

Multicomponent, behavioral intervention is the first-line standard of care for treating adolescents with obesity [1,2]. A fundamental component of this treatment approach is intensive dietary guidance to yield an energy imbalance for weight loss [2]. This dietary guidance often includes daily energy goals, with a prescription to increase low-energy-dense, nutrient-rich foods (e.g., fruits and vegetables) and decrease consumption of energy-dense, nutrient-poor items (e.g., sugar-sweetened beverages) [3]. Yet, longstanding systematic barriers and economic inequities make adherence to this dietary guidance more challenging for under-resourced populations. Families with fewer economic resources often experience food insecurity, defined by limited or uncertain access to adequate and safe foods [4]. Food insecurity presents a multitude of challenges to adopting healthful dietary patterns, including less availability, affordability, and accessibility of nutrient-rich foods [5]. Despite these widely known social and economic barriers, few investigations have examined how food insecurity is associated with adolescents’ ability to make dietary changes during obesity treatment, or if treatment response differs based on food security status.

The home food environment is one of the most salient settings for shaping children’s dietary intake [6,7]; thus, pediatric obesity treatment often includes guidance to modify the home food environment by removing energy-dense, nutrient-poor items, and making nutrient-rich items more available and accessible. Families with food insecurity lack equitable resources to make changes to their home food environment, including reduced access to nutrient-rich foods [8], as well as fewer financial resources to purchase healthier (and often more costly) foods [9] and cooking equipment [10]. While speculative, families with food insecurity may also be hesitant to purchase novel, more healthful foods that could be disliked and discarded, and less likely to remove existing energy-dense, nutrient-poor foods and beverages from their home, given concern around wasting the financial resources used to purchase these food and beverage items. These factors pose significant challenges to making positive changes in the home food environment and may impair improvements in dietary quality during treatment. While observational research has quantified links between food insecurity, lower dietary quality, and more obesogenic home food environments [11,12], there is a lack of research examining the role of food insecurity on dietary changes made during behavioral weight loss interventions for adolescents.

Food insecurity has been linked to greater obesity risk in adults, particularly in women [11,13,14]; thus, recent studies have examined the moderating role of food insecurity on weight loss during adult lifestyle interventions [15,16]. For example, Meyers et al. [16] showed that adults with food insecurity had less weight loss than food secure adults during an intensive lifestyle obesity treatment, while Berkowitz et al. [15] observed similar weight loss in adults with type 2 diabetes, regardless of food security status. During the critical developmental period of adolescence, obesity prevalence is higher among those living in food insecure households [17]; thus, research is needed to examine the role of food insecurity during obesity treatment to provide a better understanding of the extent to which food insecurity might be a barrier to achieving optimal treatment benefits for adolescents. These data can inform the development of targeted pediatric obesity treatment strategies to reduce systematic barriers to adopting healthful dietary changes and optimize weight loss outcomes.

The aim of this paper is to examine whether food security is associated with changes in the home food environment, dietary intake, and body mass index (BMI) during a behavioral weight loss intervention for adolescents with obesity. Data were from the TEENS+ intervention during which adolescents participated in a 4-month behavioral weight loss treatment. Previous reports from this study showed that adolescents in TEENS+ decreased their energy intake, improved their diet quality, and experienced significant reductions in BMI post-treatment [18,19]. The aims of this secondary analysis were to quantify changes in (1) the home food environment, (2) dietary quality, (3) daily energy intake, and (4) BMI from baseline to post-treatment by household food security status. The second aim was to examine if associations between changes in the home food environment and parallel changes in dietary quality and energy intake differed by food security status. We hypothesized adolescents in food insecure households would have less optimal diet quality, home food environments, and BMI changes during treatment, compared to adolescents in food secure households.

## 2. Materials and Methods

### 2.1. Design

TEENS+ was a 2-arm pilot randomized clinical trial, conducted from 2016–2018 in Central Virginia. Both adolescents and an adult parent or caregiver participated TEENS+—a 4-month behavioral weight loss intervention [18]. Adolescent-parent dyads were randomized into TEENS + PAC (Parents as Coaches) or TEENS + PWL (Parent Weight Loss). Study groups differed based on the parent intervention, whereas the adolescent intervention was identical in both study arms. Intervention content was delivered via weekly, in-person group sessions for 16 total weeks. Assessments occurred at baseline and post-treatment (4-months). Full study details are described in Bean et al. [18]. All study procedures were registered on clinicaltrials.gov (accessed on 22 February 2022) (#NCT02586090) and approved by the Institutional Review Board at Virginia Commonwealth University.

### 2.2. Participants

Families were recruited primarily through pediatrician referrals and mailed postcards. Interested families completed an online screening questionnaire to assess eligibility. Adolescents had to be 12–16 years of age, with a BMI ≥ 85th percentile for age and sex according to the nationally representative referent population used in the Center for Disease Control growth carts [20], and primarily residing in the participating parent’s home. Adolescents were excluded if they had a clinical eating disorder (e.g., anorexia nervosa or bulimia nervosa), significant psychopathology (e.g., suicidality), were unable to follow the study protocol due to a physical or developmental disorder, had medication changes in the past 3 months that could impact weight (e.g., antidepressants), used atypical antipsychotics, and/or were diagnosed with diabetes mellitus or other medical conditions known to impact weight (e.g., Prader Willi Syndrome). Parents had to be ≥18 years of age with a BMI ≥ 25 kg/m^2^ to be eligible. Exclusion criteria for parents mirrored that of adolescents; the only exception was that parents remained eligible if they had diabetes mellitus and were on a stable dose of diabetes medications over the past 3 months. Eligible families attended a group orientation where eligibility was confirmed, and written consent and assent were obtained. Adolescents and parents both received medical clearance to participate. A total of *n* = 82 dyads completed baseline measures and were subsequently randomized into one of the two study groups.

### 2.3. TEENS+ Intervention

All adolescents, regardless of study group, participated in the TEENS+ intervention. Manualized sessions included core evidence-based behavioral strategies around energy balance behaviors, such as goal setting, self-monitoring, contingency management, and stimulus control [21,22]. This intervention was informed by Social Cognitive Theory [23] as well as Self-Determination Theory through the use of motivational interviewing [24].

#### 2.3.1. Weekly Group Sessions

Adolescents participated in 1-h same-sex group sessions led by trained behavior coaches (psychology doctoral student, dietitians, or similar). Weight was measured before each group session during a brief individual check-in with their coach. Group sessions included a review of progress, group problem solving, and psychoeducation, with personalized goal setting and implementation planning. Adolescents kept weekly food and physical activity logs, and coaches provided personalized feedback using a self-regulation framework [25]. Adolescents also participated in 1 h of supervised exercise training on their group night in the program gym. During the final month of treatment, adolescents were encouraged to self-weigh once a week on the morning of their group night to assist in applying the self-regulation process independently.

#### 2.3.2. Individual Sessions, Cooking Class, and Exercise Opportunities

Adolescents attended a monthly individual visit with their behavioral coach or dietitian. Individual sessions used a motivational interviewing approach to explore adolescent’s personal values and reasons for behavior change and provide personalized dietary modifications to meet their weight loss goals. Adolescents were also invited to exercise at the program gym on additional days, outside of their weekly group night. Parents and adolescents were invited to attend a cooking class during treatment and were offered a local YMCA membership throughout the study duration.

#### 2.3.3. Dietary Intervention Guidance

The dietary guidance provided in TEENS+ was designed to reduce adolescents’ daily caloric intake and improve dietary quality by adding low-calorie, nutrient-dense foods (“Go Foods”), and reducing energy-dense, nutrient-poor foods [18,19,26,27]. At the start of treatment, adolescents were prescribed a daily calorie range (1200–1400 kcal/day for girls; 1500–1800 kcal/day for boys) and a Go-Food goal (# Go-Foods/day), designed to result in a 1–2 lb weight loss each week. Daily food logs were used to track dietary intake and self-monitor adolescents’ alignment with these goals. Calorie goals were adjusted throughout treatment, as needed, and Go-Food goals were increased once previous goals were met. Dietary education included lessons on energy balance behaviors and ways to improve dietary quality, such as reducing sugar-sweetened beverages, creating healthy meal patterns, and consuming more foods at home rather than eating out.

### 2.4. Parent Intervention

The parent interventions are described in full elsewhere [18]. Parents participated in their assigned intervention (PAC or PWL), via weekly group sessions led by trained behavioral coaches on the same night as their adolescent’s group session.

#### 2.4.1. Parents as Coaches

PAC taught parent skills training and nutrition education to emphasize parents’ role in assisting adolescents with weight loss. Parents were taught authoritative parenting through role modeling and providing healthful food and exercise opportunities. Guidance on modifying the home food environment included removing energy-dense, nutrient-poor items, replacing these with nutrient-rich foods, and making them easily accessible and visible. Parenting behaviors were logged each week, and coaches provided personalized feedback. Parent weight loss was not addressed or monitored in this study arm.

#### 2.4.2. Parent Weight Loss

PWL focused on parents’ concurrent engagement in lifestyle behaviors to support their own weight loss. Parents received calorie, fat, and physical activity goals, designed to result in a 1–2 lb weight loss each week. Parents were encouraged to consume nutrient-rich foods and decrease their consumption of nutrient-poor, energy-dense items to achieve a calorie deficit and improve diet quality. Parent weight was measured each week during group sessions, and parents were encouraged to self-weigh daily. Dietary intake, weight, and exercise were logged daily with personalized feedback provided. Key behavioral weight loss strategies were taught to help parents meet their weight loss goals [28], including use of stimulus control strategies (e.g., modifying the home food environment) to support their weight loss goals. PWL did not contain guidance around parenting to support adolescent weight loss.

### 2.5. Retention and Fidelity Protocols

Engagement, retention, and fidelity strategies are detailed elsewhere [18]. Briefly, raffle tickets were provided for attendance and completion of self-monitoring logs. Raffle winners were selected at variable intervals, and monthly group incentives were provided to all adolescents. Coaches contacted families that missed group sessions and provided lesson materials via email. Compensation of $40 was provided for completing the post-treatment assessment visits. All behavioral coaches were masked to study hypotheses. Coaches were trained following a standardized operations manual that included study arm-specific content and strategies for culturally informed intervention delivery [29]. Coaches conducted mock treatment sessions before intervention delivery and received detailed feedback from lead supervisors. All treatment sessions were audio recorded and reviewed in weekly supervision.

### 2.6. Measures

Assessments were conducted at baseline and post-treatment. Surveys were completed online via REDCap [30]. Dietary assessments and anthropometrics were completed in-person by trained assessors masked to study hypotheses.

#### 2.6.1. Food Insecurity

Parents completed the 6-item United States Department of Agriculture Household Food Security Module at baseline and post-treatment to quantify household food security status at each timepoint [31]. This brief version was chosen to minimize participant burden, while maintaining high specificity and sensitivity with minimal bias [32]. Affirmative responses were totaled, and families were categorized as food secure (scores of 0–1) or food insecure (scores of 2–6) at baseline and post-treatment.

#### 2.6.2. Home Food Environment

Parents completed the home food inventory (HFI) [33] at baseline and post-treatment to assess the foods and beverages available in their home. This 190-item questionnaire listed foods and beverages in a checklist format, and parents selected if each item was present or not in their home (yes (1), no (0)). An obesogenic food availability score was calculated by summing the affirmative responses for 10 subscales (regular fat cheese, milk, yogurt, and other dairy; processed meat; regular frozen dessert and prepared desserts; high-sugar cereal; candy; and microwaveable or quick-cook food) and 22 items from the categories of added fats, savory snacks, beverages, access to unhealthy foods in the kitchen, and access to unhealthy foods in the refrigerator [33]. Lower scores indicated a less obesogenic home environment. The HFI has been validated in populations similar to the present study, and the obesogenic food availability score has been associated with parent and adolescent energy intake [33].

#### 2.6.3. Dietary Intake

Adolescent dietary intake was assessed via 3-day food records at baseline and post-treatment. Adolescents used paper logs to record their food and beverage intake across 2 typical weekdays and 1 typical weekend day. Dietary records were reviewed in-person with a trained study dietitian where additional details were obtained (e.g., cooking method, brands, amount consumed) using food models and portion size guides. Food record data were entered into Nutrition Data Systems for Research (NDSR; Nutrition Coordinating Center, University of Minnesota, Minneapolis, Minnesota), and daily energy intake was derived by calculating the average daily calories consumed across all three days.

The Healthy Eating Index 2015 (HEI-2015) was used to assess dietary quality at each timepoint [34]. The HEI-2015 is comprised of 13 subscales including: total fruits, whole fruits, total vegetables, greens and beans, whole grains, dairy, total protein foods, seafood and plant proteins, fatty acids (i.e., ratio of poly- and mono-unsaturated fatty acids to saturated fatty acids), refined grains, sodium, added sugars, and saturated fats. HEI total scores range from 0 to 100, with higher scores indicating more healthful dietary intake. Two composite subscores were also calculated according to the procedures detailed in Raynor et al. [19]. The “Increase” subscore included nine components of the HEI in which increased intake is recommended (total fruits, whole fruits, total vegetables, greens and beans, whole grains, dairy, total protein foods, seafood and plant proteins, and fatty acids). Possible scores ranged from 0 to 60, with higher scores indicating greater consumption of these nutrient-rich foods. The “Decrease” subscore contained four components of the HEI in which consumption in moderation is recommended (refined grains, sodium, added sugars, and saturated fats). Possible scores ranged from 0 to 40, with a higher score indicating lower consumption of these nutrient-poor, energy-dense foods.

#### 2.6.4. Demographics

At baseline, parents reported on adolescent age, sex, race, ethnicity; household income; and insurance status.

#### 2.6.5. Anthropometrics

Three weight measurements were obtained using an electronic digital scale (Scale-Tronix 5125-X, Welch Allyn, Aurburn, NY, USA), after a 12-h fast and when wearing light clothing. Values were rounded to the nearest 0.1 kg. Three height measurements were obtained using a wall-mounted stadiometer (Seca 213), with values rounded to the nearest 0.1 cm. Adolescent BMI (kg/m^2^) was calculated using the average of these three measurements and BMI percentile was determined using Epi Info Software [35]. Changes in BMI across treatment were quantified as post-treatment minus baseline values.

### 2.7. Statistical Analyses

Descriptive statistics were used to quantify participant demographics. All analyses combined data across both intervention study groups, given all adolescents received the same intervention, and no between-group differences in adolescent weight loss were observed [18]. All analyses also used a conservative intent-to-treat (ITT) approach, wherein non-completers at post-treatment (*n* = 12) were assumed to have returned to baseline values by post-treatment. Food security was calculated at baseline and post-treatment to describe the extent to which families remained stable or transient in food security over time. Few families changed food security status across treatment in this sample; thus, food security status at baseline was used for all subsequent analyses to address the study aims.

For Aim 1, repeated measures analysis of variance models using PROC MIXED examined patterns in the home food environment, dietary quality, daily energy intake, and BMI from baseline to post-treatment. Given the smaller sample size of this pilot study, models were stratified by baseline household food security status to examine patterns across treatment for food secure and food insecure families separately. All models controlled for intervention study group. Baseline and post-treatment values are presented as unadjusted means ± standard deviations. Mean difference values (post-treatment minus baseline) ± standard deviations are also reported for descriptive purposes.

For Aim 2, change score variables were calculated (post-treatment minus baseline) for changes in the home food environment, dietary quality, daily energy intake, and BMI across treatment. Negative change score values indicate positive changes to the home food environment, daily energy intake, and BMI; positive change score values indicated positive changes to dietary quality variables across treatment. One outlier for changes in the home food environment across treatment was identified, with a change score value >3 standard deviations above the mean. To retain all participants, winsorization was applied by identifying the next closest value for this variable and applying this value to the outlier’s mean difference. Next, the degree of change for the windsorized mean difference was reflected in the post-treatment value for this same participant to maintain consistency. [36]. Linear regression models were used to examine if change score values in the home food environment were associated with changes in dietary quality, daily energy intake, and BMI. Similar to Aim 1, all models were stratified by baseline food security status. Data are presented two ways, as raw data with associated beta coefficients (*B*) and standard errors (SE), as well as in models controlling for intervention study group. *p* values < 0.05 were considered significant. All analyses were performed in SAS Studio version 3.8.

## 3. Results

Adolescent and household demographics are presented by food security status in Table 1. Adolescents were mostly female (63%) and 13.7 ± 1.2 years of age. Approximately half (46%) were Black, and 50% were non-Hispanic White. Most families (76%) had private insurance, while 60% had a household income of ≤$75,000/year. About one-fourth (23%) of families reported food insecurity at baseline, and 24% of families reported food insecurity at post-treatment.

### 3.1. Patterns in the Home Food Environment, Adolescent Dietary Intake, and BMI across Treatment by Food Security Status

Households that were food secure showed improvements in their home food environment across treatment, while households that were food insecure showed no changes (HFI obesogenic home food availability score mean difference (post-treatment minus baseline): −6.6 ± 6.4 vs. −2.4 ± 7.4, respectively; Table 2). Adolescents in food secure households had improved overall diet quality and increased consumption of healthful dietary components, while adolescents in food insecure households showed no change (HEI total score, mean difference: 5.1 ± 14.4 vs. 2.7 ± 17.7, respectively; HEI Increase subscore, mean difference: 3.7 ± 9.0 vs. 2.0 ± 10.7, respectively). Adolescents’ consumption of unhealthful dietary components did not change across treatment, regardless of food security status (HEI Decrease subscore, mean difference: 1.3 ± 6.6 vs. 0.8 ± 8.1, respectively). Daily energy intake and BMI decreased for adolescents in both food secure and insecure households across treatment (daily energy intake, mean difference: −287 ± 417 kcal/day vs. −309 ± 434 kcal/day; BMI, mean difference: −1.0 ± 1.4 vs. −0.7 ± 1.4, respectively).

### 3.2. Associations between Changes in the Home Food Environment with Adolescents’ Dietary Intake across Treatment

**Figure 1:**
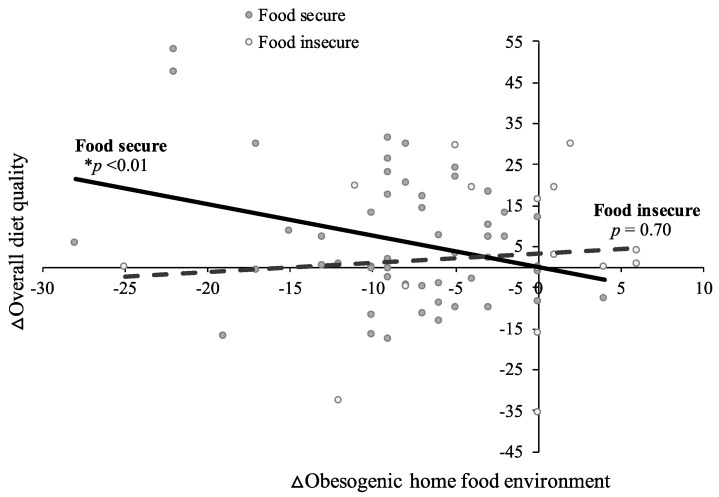
Positive improvements in obesogenic home food availability (as indicated by negative change score values) were associated with positive changes in overall diet quality (as indicated by positive change score values) for adolescents in food secure (*p* = 0.01), but not food insecure (*p* = 0.88), households. Changes were measured from baseline to post-treatment during a 4-month behavioral weight loss intervention among *n* = 82 adolescents with overweight or obesity. Raw data points and associated regression lines are presented. * *p* < 0.05; ∆ indicates change score values (post-treatment minus baseline).

**Figure 2:**
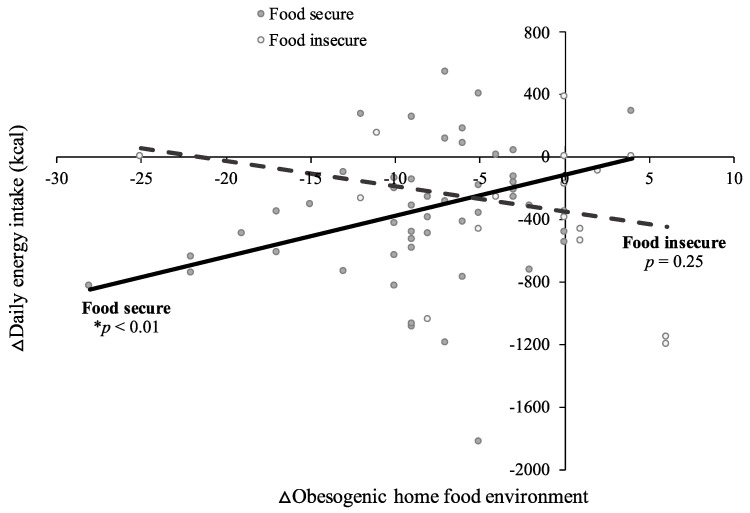
Positive improvements in obesogenic home food availability (as indicated by negative change score values) were associated with positive changes in daily energy intake (as indicated by negative change score values) for adolescents in food secure (*p* < 0.01), but not food insecure (*p* = 0.25), households. Changes were measured from baseline to post-treatment during a 4-month behavioral weight loss intervention among *n* = 82 adolescents with overweight or obesity. Raw data points and associated regression lines are presented. * *p* < 0.05; ∆ indicates change score values (post-treatment minus baseline).

**Figure 3:**
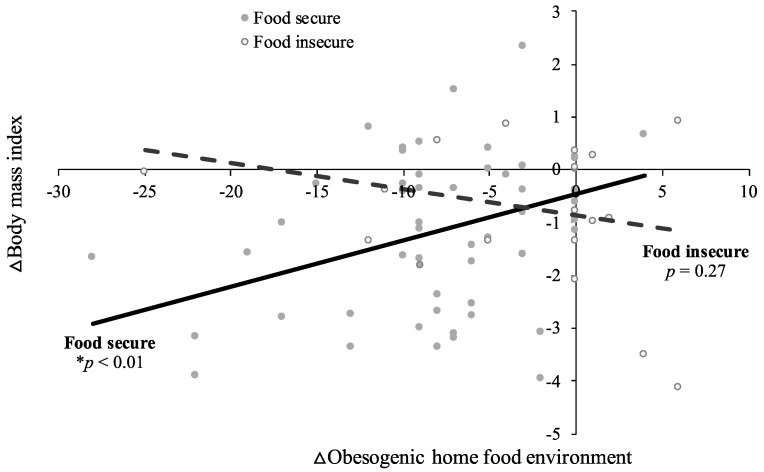
Positive improvements in obesogenic home food availability (as indicated by negative change score values) were associated with positive changes in body mass index (as indicated by negative change score values) for adolescents in food secure (*p* < 0.01), but not food insecure (*p* = 0.30), households. Changes were measured from baseline to post-treatment during a 4-month behavioral weight loss intervention among *n* = 82 adolescents with overweight or obesity. Raw data points and associated regression lines are presented. * *p* < 0.05; ∆ indicates change score values (post-treatment minus baseline).

Associations between changes in the home food environment and dietary quality, energy intake, and BMI across treatment varied by baseline food security status. For families with food security, a reduced obesogenic home food environment predicted improved overall diet quality (B = −0.7; SE = 0.3; *p* = 0.01; Supplementary Table S1), increased consumption of healthful dietary components (HEI Increase subscore: B = −0.5; SE = 0.2; *p* < 0.01), reduced daily energy intake (B = 26.1; SE = 7.8; *p* < 0.01; Supplementary Table S1), and reduced BMI (B = 0.1; SE = 0.03; *p* < 0.01; Supplementary Table S1) across treatment, when controlling for study group. These relations were not observed for families with food insecurity (HEI total score: B = 0.1; SE = 0.6; *p* = 0.88; HEI Increase subscore: B = −0.1; SE = 0.4; *p* = 0.86; daily energy intake: B = −17.0; SE = 14.3; *p* = 0.25; BMI: B = −0.05; SE = 0.05; *p* = 0.31; Supplementary Table S1). There was no association between changes in the home food environment and consumption of unhealthful dietary components, regardless of food security status (HEI Decrease subscore; food secure: B = −0.2; SE = 0.1; *p* = 0.14; food insecure: B = 0.1; SE = 0.3; *p* = 0.57). Similar patterns emerged when examining patterns in the raw data (Figure 1, Figure 2 and Figure 3).

## 4. Discussion

This study described changes in adolescents’ home food environment, dietary quality, energy intake, and BMI across behavioral weight loss treatment by household food security status. Main findings indicated that BMI and energy intake reductions across treatment were observed for both food secure and food insecure adolescents; however, significant improvements to the home food environment and dietary quality were only observed in adolescents from food secure families. Reductions in obesogenic home food environments were associated with improved diet quality, reduced energy intake, and reduced BMI for food secure families, yet these associations were not present for food insecure households. Collectively, these findings highlight food insecurity as a barrier to the successful adoption of nutrient-rich dietary patterns and positive home food environment changes during adolescent obesity treatment.

Adolescents in TEENS+ who lived in food secure—but not food insecure—households made positive changes to dietary quality and the home food environment across treatment. Specifically, adolescents in food secure households increased consumption of nutrient-rich, low energy-dense dietary components, while adolescents in food insecure households did not adopt these same changes. In accordance with pediatric behavioral weight loss recommendations [3], central themes to TEENS+ included strategies to increase home food availability and consumption of nutrient-rich foods, and decrease the availability and consumption of energy-dense, nutrient-poor items. For adolescents in food secure households, a reduced obesogenic home food environment was associated with improved diet and weight outcomes; yet, for adolescents in food insecure households, these associations were not observed. This difference is likely due to the less robust changes to the home food environment for food insecure families, where financial, structural, and environmental challenges [5] likely presented barriers to the adoption of healthful dietary behaviors. 

Gold standard behavioral weight loss treatment focuses on building knowledge and skills around dietary behavior change. Yet, to facilitate the adoption of this guidance, it is important to consider if additional strategies to reduce barriers to healthful food access are needed to increase the efficacy of this treatment approach, particularly for families with food insecurity. This could involve the translation of guidance from organizations such as the American Academy of Pediatrics [37], American Diabetes Association [38], and American Psychological Association [39] into obesity treatment settings by screening for food insecurity at the start of treatment and connecting families with food resources that aid in food access (e.g., local food banks, Supplemental Nutrition Assistance Program). Connecting families to existing community resources capitalizes on sustainable methods for reducing food insecurity; yet efforts will be needed to ensure families overcome prominent barriers to accessing these food resources (e.g., transportation, difficulty with enrollment) and obtain healthful foods that improve diet quality. Another approach could involve fruit and vegetable prescriptions as a component of obesity treatment, similar to recent approaches implemented within routine clinical care [40]. Fruit and vegetable prescriptions may provide a low-risk opportunity to expose families to nutrient-rich foods that improve diet quality; yet this strategy may not serve as a long-term sustainable solution for improving nutrition security.

Future research is needed to identify if these, or similar, strategies improve healthy food access and can be implemented within behavioral weight loss treatment in a manner that is well-received and sustainable. Clinical trials are needed to examine if the incorporation of these strategies reduces food access barriers and results in greater improvements to dietary changes during, and after, obesity treatment. Public health policies related to food access and availability are also instrumental in shaping population-level impacts on dietary outcomes. Despite being external to the individual treatment context, these policies are critical to treatment success [41]. The current obesogenic environment provides significant challenges to accessing, adopting, and maintaining healthy dietary behaviors [42]. Thus, coordinated efforts across public health sectors and clinical care settings are needed for effective obesity treatment that results in sustained improvements to dietary and weight outcomes. Historically, public health policies and clinical care efforts have focused on reducing food insecurity by providing families with access to sufficient calories and quantities of food; yet, thoughtful efforts are needed to ensure these initiatives also promote nutrition security, by increasing access to nutrient-rich foods that improve diet quality and lower obesity and chronic disease risk [43]. 

Obesity is influenced by a multitude of environmental, biological, social, behavioral, and genetic factors [44]; yet a fundamental component of obesity treatment includes creating an energy imbalance to facilitate weight loss. Changes in daily energy intake across treatment were examined by food security status, finding that adolescents, regardless of food security status, decreased energy intake by ~300 kcal/day. This caloric deficit likely contributed to a decrease in BMI across treatment for both food secure and insecure adolescents. This finding indicates that, despite an absence of improved dietary quality, adolescents in food insecure households were able to achieve significant weight loss, likely due to this energy deficit. These patterns differ from those observed in adults, where food secure adults lost approximately twice as much weight during an intensive lifestyle intervention, compared to adults with food insecurity [16]. Findings from this study are positive in that adolescent weight loss was achieved during treatment for both food secure and insecure families; yet, potential detriments to not improving diet quality for food insecure families must also be realized [45]. Less robust changes to adolescents’ dietary quality and the home food environment may pose significant barriers for weight loss maintenance and overall chronic disease risk, which should be examined in future investigations.

A limitation of this pilot study includes the small sample size with data stratified by food security status. This allowed for the quantification of within-group patterns; yet not between-group differences, and findings should be interpreted as preliminary patterns to be examined in a larger trial. The 6-item USDA household food insecurity module was used to minimize participant burden [31], yet this version does not distinguish between adult and child food insecurity. It is thus uncertain if adolescents living in food insecure households were experiencing food insecurity themselves. As an alternative, the 18-item USDA food insecurity measure [46] could be used, when feasible, to quantify child food insecurity more specifically. Finally, food insecurity was measured at baseline and post-treatment, yet, not periodically throughout treatment. While only five families in this sample transitioned between food security and insecurity across treatment, the broader literature suggests a cyclical and transient nature of food insecurity for many families [47]. Thus, when feasible, routine measurement of food insecurity should be conducted to examine temporal patterns in food insecurity with dietary and weight outcomes. Despite these limitations, this study is significantly strengthened by the rigorous measurement of dietary and weight measures and the focus on adolescents who are an understudied population in the area of pediatric obesity treatment.

## 5. Conclusions

This study is one of the first investigations to examine dietary and weight outcomes by food security status during adolescent obesity treatment. This behavioral weight loss program for obesity treatment yielded similar reductions in energy intake and weight for all families, yet it did not appear to offer the same treatment benefits regarding improved diet quality and home food environments for families with food insecurity. Future behavioral weight loss programs should consider the addition of food insecurity measurement and the incorporation of targeted strategies to reduce barriers to healthful food access and affordability for food insecure families.

## Figures and Tables

**Table 1:** Baseline demographics of adolescents (*n* = 82) enrolled in TEENS+ by baseline household food security status.

	Food Secure (*n* = 63)	Food Insecure (*n* = 19)
Female, *n* (%)	39 (62)	13 (68)
Race ^a^, *n* (%)		
African American/Black	24 (38)	14 (74)
White	38 (60)	3 (16)
Asian	3 (5)	0 (0)
Native American	1 (2)	1 (5)
Other	3 (5)	2 (11)
Hispanic, *n* (%)	3 (5)	1 (5)
Family insurance status ^b^, *n* (%)		
None	1 (2)	1 (6)
Medicaid	11 (18)	6 (33)
Private Insurance	50 (81)	11 (61)
Annual family income ^b^, *n* (%)		
<$10,000	1 (2)	1 (6)
$10,000–19,999	3 (5)	4 (22)
$20,000–29,999	6 (10)	2 (11)
$30,000–39,999	3 (5)	3 (17)
$40,000–49,999	1 (2)	1 (6)
$50,000–74,999	19 (30)	4 (22)
$75,000–99,999	13 (21)	0 (0)
$100,000–149,999	13 (21)	3 (17)
>$150,000	3 (5)	0 (0)
Total individuals living in the home	4.1 ± 1.0	3.9 ± 1.0
Child:adult ratio living in the home	1.0:1	1.4:1
Age (years), mean (SD)	13.7 ± 1.2	13.6 ± 1.3
Weight (kg), mean (SD)	93.4 ± 19.3	97.4 ± 19.9
BMI (kg/m^2^), mean (SD)	34.5 ± 6.8	36.1 ± 7.6
BMI percentile, mean (SD)	98.3 ± 1.3	98.4 ± 1.7

^a^*n* = 2 adolescents declined to provide race information; participants could select more than 1 racial category; thus, percentages do not total 100%; ^b^ parent-reported at baseline. BMI = body mass index.

**Table 2:** Changes in the home food environment, adolescent dietary quality, and daily energy intake during TEENS+, a 4-month behavioral weight loss intervention for obesity treatment. Values presented for adolescents (*n* = 82 total) in households that were food secure vs. insecure at baseline.

	Food Secure ^a^ (*n* = 63)			Food Insecure ^a^ (*n* = 19)		
	Baseline	Post-Treatment	*p* Value ^g^	Baseline	Post-Treatment	*p* Value ^g^
Obesogenic home food availability ^b^	19.3 ± 8.5	12.7 ± 8.9	<0.01	14.9 ± 8.8	12.5 ± 9.1	0.18
Healthy Eating Index (HEI)-2015						
Total score ^c^	50.2 ± 12.8	55.2 ± 13.2	<0.01	53.7 ± 12.9	56.4 ± 13.7	0.51
Increase component ^d^	30.3 ± 8.7	34.1 ± 8.9	<0.01	33.4 ± 7.8	35.3 ± 8.8	0.43
Decrease component ^e^	19.8 ± 5.4	21.2 ± 5.6	0.11	20.3 ± 5.7	21.1 ± 5.9	0.68
Daily energy intake (kcal/day) ^f^	1797 ± 509	1510 ± 456	<0.01	1659 ± 502	1350 ± 466	<0.01
Body mass index (kg/m^2^)	34.5 ± 6.8	33.4 ± 7.2	<0.01	36.1 ± 7.6	35.4 ± 8.0	0.03

^a^ Measured at baseline; ^b^ from the home food inventory; lower scores = less obesogenic home food environment; ^c^ possible range = 0–100; higher scores = greater dietary quality; ^d^ sub-components of HEI to increase consumption; possible range = 0–60; higher scores = greater consumption of healthful foods; ^e^ sub-components to HEI to decrease consumption; possible range = 0–40; higher scores = less consumption of unhealthful foods; ^f^ average daily energy intake derived from 3-day food records; ^g^ indicate baseline to post-treatment differences when data are stratified by food security status category.

## Data Availability

Following the publication of associated research findings, M.K.B. will accept data-sharing requests from qualified investigators within the greater scientific community. All requests for data sharing will be reviewed and approved by the M.K.B. prior to the release of data. Shared datasets and corresponding data dictionaries would be free of identifiers or variables that would permit linkage to or lead to deductive disclosure of the identity of individual subjects. All data-sharing procedures would be in compliance with institutional and IRB policy at Virginia Commonwealth University, NIH policy, HIPAA, and other local, state, and federal laws and regulations.

## Supplementary Materials

The following supporting information can be downloaded at: https://www.mdpi.com/article/10.3390/nu14050976/s1, Table S1: Associations between changes in the obesogenic home food environment with adolescents’ dietary quality, daily energy intake, and body mass index from baseline to post-treatment, following a 4-month multicomponent obesity intervention in *n* = 82 adolescents. Test statistics are from linear regression models stratified by baseline food security status and controlling for intervention study group.
