# Supplementary material for: Home Food Environment Changes and Dietary Intake during an Adolescent Behavioral Weight Loss Intervention Differ by Food Security Status
Source: Nutrients. 2022 Feb 25;14(5):976. doi: 10.3390/nu14050976 (PMC8912871; doi:10.3390/nu14050976)
Supplement: Supplementary file 1 [file nutrients-14-00976-s001.zip › nutrients-1591386-supplementary.pdf]

Supplementary Table S1. Associations between changes in the obesogenic home food environment with adolescents' dietary quality, daily energy intake, and body mass index from baseline to post-treatment, following a 4-month multicomponent obesity intervention in N=82 adolescents. Test statistics are from linear regression models stratified by baseline food security status and controlling for intervention study group.

	△Obesogenic home food availability[a]			
	Intercept	Beta coefficient	Standard error	F value (p value)
**Model 1: △Diet quality[b]**				
Food secure	-2.31	-0.70	0.27	6.90 (0.01)
Food insecure	-3.93	0.09	0.58	0.02 (0.88)
**Model 2: △Daily energy intake[c]**				
Food secure	-111.0	26.10	7.78	11.26 (<0.01)
Food insecure	-388.74	-17.04	14.33	1.41 (0.25)
**Model 3: △Body mass index[d]**				
Food secure	-0.54	0.09	0.03	12.03 (<0.01)
Food insecure	-0.78	-0.05	0.05	1.11 (0.31)

△ values calculated as post-treatment minus baseline

aMeasured with the Home Food Inventory; Lower scores = less obesogenic home food environment

bMeasured with the Healthy Eating Index. Higher scores = better dietary quality

cMeasured in kcal/day

dMeasured in kg/m2
